# Mechanisms of the immunosuppressive effects of mouse adipose tissue-derived mesenchymal stromal cells on mouse alloreactively stimulated spleen cells

**DOI:** 10.3892/etm.2013.1382

**Published:** 2013-11-06

**Authors:** RYO NAGAYA, MASAKO MIZUNO-KAMIYA, EIJI TAKAYAMA, HARUMI KAWAKI, IPPEI ONOE, TOSHIICHIRO TANABE, KUNITERU NAGAHARA, NOBUO KONDOH

**Affiliations:** 1 Department of Oral Implantology, Asahi University School of Dentistry, Mizuho-shi, Gifu 501-0296, Japan; 2 Department of Oral Biochemistry, Asahi University School of Dentistry, Mizuho-shi, Gifu 501-0296, Japan

**Keywords:** immunosuppression, adipose tissue-derived stromal cells, β2 microglobulin, interferon-γ

## Abstract

The mechanisms of immunomodulation by mesenchymal stromal cells remain poorly understood. In this study, the effects of mouse adipose tissue-derived mesenchymal stromal cells (ASCs) on mouse spleen cells alloreactively stimulated by anti-CD3 and anti-CD28 antibody-coated (anti-CD3/CD28) beads were observed. Production of interferon-γ by the anti-CD3/CD28 bead-stimulated spleen cells was significantly suppressed in co-culture with ASCs. However, an augmented intensity of CD69 on the stimulated spleen cells was not suppressed in the presence of ASCs. The immunosuppressive effects of ASCs were partially mediated by one or more soluble factors (26% suppression). However, the ASCs require cell-cell contact in order to maximally exert suppression (88%). The suppressive effect of ASCs mediated by direct cell contact was partially reversed following knockdown of β2 microglobulin, a component of the major histocompatibility complex (MHC) class I molecule, with siRNA. The results of the study demonstrated that ASCs have significant immune modulatory effects on alloreactively stimulated spleen cells. The effects of ASCs on spleen cells are dependent on soluble factor(s) and cell contact, which is mediated by the MHC class I complex on ASCs.

## Introduction

Mesenchymal stromal cells (MSCs) are originally isolated from bone marrow (BM) (1). MSCs show proliferation without the loss of undifferentiated phenotype and retain the ability to differentiate into several mesenchymal lineages, such as bone, cartilage, adipose and muscle tissues (2). In addition to BM, MSCs have also been isolated from adipose tissue (3), placenta (4), amniotic fluid (5) and fetal tissues (6). The percentage of MSCs in BM is low (0.001-0.01% of the mononuclear cell fraction). By contrast, adipose tissue contains a ~500-fold percentage of MSCs than BM and the process of tissue collection is simple (7).

An important characteristic of MSCs is their immunomodulatory capacity. MSCs suppress the proliferation of T cells upon allogeneic (8–19) or mitogenic stimulation (11), promote apoptosis of activated T cells (12) and enhance the generation of regulatory T cells (13). MSCs also inhibit the proliferation of B cells and natural killer cells (14,15). Several factors have been implicated in the immunomodulatory effects of MSCs, including prostaglandin E2 (PGE_2_), transforming growth factor-β1 (TGF-β1) and indoleamine 2,3-dioxygenase (IDO) (15,16). In experimental models, administration of MSCs resulted in the prevention of graft-versus-host disease (GvHD) (17) in prolonged skin graft survival (18). The use of MSCs as a cellular therapy has been examined in clinical trials to treat GvHD (19) and Crohn’s disease (20). MSCs express intermediate levels of major histocompatibility complex (MHC) class I molecules and very low levels of class II (21), which may be recognized by alloreactive T cells. Notably, the immunomodulatory capacity of adipose tissue-derived mesenchymal stromal cells (ASCs) is higher than that of BM-derived MSCs (22).

In the present study, the direct and indirect effects of ASCs on alloreactively stimulated mouse spleen cells were observed. In addition, the interferon-γ (IFN-γ)-producing capability of the spleen cells, the population of activated CD69^+^ cells among CD45^+^ leukocytes and the functions of MHC molecules on ASCs were investigated.

## Materials and methods

### Experimental animals

Thirty male BALB/c mice were purchased from Chubu Kagaku Shizai Co., Ltd. (Nagoya, Japan) and had access to Oriental MF solid chow (Oriental Yeast Co., Tokyo, Japan) and water *ad libitum*. This study was approved by the Animal Ethics Committee of Asahi University (Gifu, Japan; grant no. 07–016).

### Harvest and primary culture of ASCs

Four-week-old male BALB/c mice (weight, 15–20g) were sacrificed by cervical dislocation. The inguinal fat pads were harvested and washed with phosphate-buffered saline (PBS). They were excised, finely minced and then digested with 0.1% collagenase (Wako Pure Chemical Industries, Ltd., Osaka, Japan) for 40 min at 37°C. After digestion, they were filtered through a cell strainer (BD Biosciences, San Jose, CA, USA). An equal volume of starting medium (FM-start™; Toyobo Co., Ltd., Osaka, Japan) was added to the cell suspension, which was then centrifuged at 270 × g for 5 min. Cells were resuspended with 10 ml starting medium, plated on 100-mm tissue culture plates and then incubated at 37°C in 5% CO_2_. The medium was replaced every 3 days and the non-adherent cells were discarded. The cells were harvested at 80–90% confluence with 0.25% trypsin/0.1% ethylenediaminetetraacetic acid (EDTA; Invitrogen Life Technologies, Grand Island, NY, USA), collected by centrifugation at 190 × g for 5 min at room temperature, then passaged at a ratio of 1:3. The cells were cultured in FM-medium™ (Toyobo Co., Ltd.) at 37°C, 5% CO_2_. Of the cultured ASCs, passage 3 were used in this study.

### Phenotype and differentiation capacity of ASCs

The capacity of ASCs to differentiate along adipogenic and osteogenic lineages was assessed as previously described (23). Briefly, for adipogenic differentiation, cells were induced by adding 1-methyl-3-isobutylxanthine (0.5 mM), insulin (10 μM), indomethacin (200 μM) and dexamethasone (1 μM) to Dulbecco’s modified Eagle’s medium (DMEM; Wako Pure Chemical Industries, Ltd.), containing 10% fetal bovine serum (FBS; Invitrogen) and 1% antibiotic antimycotic solution (10,000 U/ml penicillin, 10,000 μg/ml streptomycin and 25 μg/ml amphotericin B; Gibco-BRL). This medium was replaced every 3–4 days for 2 weeks. Adipogenesis was measured by the accumulation of neutral lipids in fat vacuoles, observed using Oil red O staining.

For osteogenic differentiation, cells were grown in minimum essential medium-α (MEM-α; Wako Pure Chemical Industries, Ltd.,) supplemented with ascorbic acid (50 μg/ml) and glycerophosphate (10 mM) containing 10% FBS (JRH Biosciences, Lenexa, KS, USA) and 1% Pen Strep (penicillin, 10,000 U/ml and streptomycin, 10,000 μg/ml; Gibco-BRL). This medium was replaced every 3–4 days for 3 weeks. Differentiated cells were examined by Alizarin red (Wako Pure Chemical Industries, Ltd.) staining.

### Preparation of mouse spleen cells

Twenty-four-week-old male BALB/c mice were sacrificed by cervical dislocation. The spleen was removed. Spleen cells were isolated by smashing the tissue with stainless steel mesh in RPMI-1640 medium (Sigma-Aldrich, St. Louis, MO, USA) containing 10% FBS (Biowest SAS, Nuaillé, France), 50 μM 2-mercaptoethanol (Nacalai Tesque, Inc., Kyoto, Japan) and 1% antibiotic antimycotic solution (Gibco-BRL). Cells were collected by centrifugation at 430 × g for 5 min and then resuspended with 10 ml red blood cell lysis buffer [10 mM Tris-HCl (pH 7.3) containing 140 mM NH_4_Cl and 1 mM EDTA]. After incubation for 5 min at room temperature, the cells were washed three times with RPMI-1640 medium and centrifuged at 1,500 rpm for 5 min. The spleen cells were re-suspended with RPMI-1640 medium and filtered using a cell strainer (BD Biosciences) to remove the residue.

### Analysis of cytokine production by spleen cells

Spleen cells were suspended in RPMI-1640 supplemented with 10% FBS, 50 μM 2-mercaptoethanol and 1% antibiotic antimycotic solution. Cell suspension (4×10^6^/ml) was added (0.1 ml/well, in triplicate) to a 96-well plate, to which 4×10^5^ of anti-CD3 and anti-CD28 antibody-coated (anti-CD3/CD28) beads (Dynabeads^®^ Mouse T-Activator CD3/CD28; Invitrogen Life Technologies) were added. Additionally, ASCs (0–16,000 cells/well) were added to the wells. To examine the indirect effects of ASCs on the stimulated spleen cells, Transwell chambers (pore size, 0.4 μm; Corning Inc., Corning, NY, USA) 24-well plates were used. The spleen cell suspension together with anti-CD3/CD28 beads were transferred to the upper chambers and ASCs were added to the bottom chambers of a Transwell.

The cells were incubated for 48 h in 5% CO_2_ at 37°C, and then the supernatant was harvested by centrifugation at 1,710 × g for 5 min and stored at −80°C. Production of IFN-γ in the supernatant of cell culture was assayed by enzyme-linked immunosorbent assay using BD OptE1A Set Mouse IFN-γ (BD Biosciences).

### Flow cytometry

A four-colored panel was used to analyze the stimulated spleen cells. The spleen cells were harvested from the culture and transferred to a Falcon tube by thorough resuspension with a pipette. The cells were washed twice with PBS and stained with anti-mouse antibodies (mAbs), including phycoerythrin (PE)-conjugated mAb specific for CD4 (clone GK1.5), peridinin chlorophyll-a protein-cyanine 5.5 (PerCP-Cy™5.5)-conjugated mAb specific for CD45 (clone 104) and allophycocyanin (APC)-conjugated mAb specific for CD69 (clone H1.2F3; eBioscience, San Diego, CA, USA). Fluorescein isothiocyanate (FITC)-conjugated mAb specific for CD8 (KT15) was purchased from Immunotech (Marseille, France). Cells were re-suspended in PBS containing 2% FBS, 1 mM EDTA and 1% sodium azide, then analyzed by flow cytometry (FACSCalibur; BD Bioscience) with Cell Quest software (BD Bioscience).

### Knockdown of β2-microglobulin (β2M) in ASCs

β2M siRNA duplex (Silencer^®^ select Pre-designed siRNA; Ambion, Invitrogen Life Technologies) was used to knockdown the representative gene in ASCs. Sense and antisense sequences of β2M siRNA duplex were: 5′-gcc uca cau uga aau cca att-3′ and 5′-uug gau uuc aau gug agg cgg-3′. Briefly, ASCs (1×10^4^) were seeded in a 96-well plate with 0.1 ml Opti-MEM (Invitrogen Life Technologies). Then, 6 pmol siRNA and 1 μl Lipofectamine (Invitrogen Life Technologies) were added. Cells with reagents were incubated for 20 min in 5% CO_2_ at 37°C, then 10 μl FBS was added. The mixture was incubated for 4 h, and then the medium was replaced with DMEM containing 10% FBS. After 48 h, transfected ASCs were used for further experiments.

### Quantitative polymerase chain reaction (qPCR)

Knockdown of endogenous β2M in ASCs was confirmed by semi-qPCR. The whole-cell RNA extraction and semi-qPCR technique were conducted as previously described (24). Primer sequences were as follows: β2M, forward 5′-gca ggc gta tgt atc agt ctc agt-3′ and reverse 5′-gag aat ggg aag ccg aac ata ct-3′; ribosomal protein S5 (RPS5), forward 5′-aga aga ctc aac acg cat tgg gc-3′ and reverse 5′-gca ctc agc gat ggt ctt gat gt-3′. The expression levels of β2M mRNA were normalized as a ratio to that of RPS5-mRNA.

### Statistics

Data are expressed as mean ± standard deviation. Student’s t-test was applied to determine the significance of differences between two groups. P<0.05 was considered to indicate a statistically significant difference.

## Results

### In vitro differentiation of ASCs

**Figure 1:**
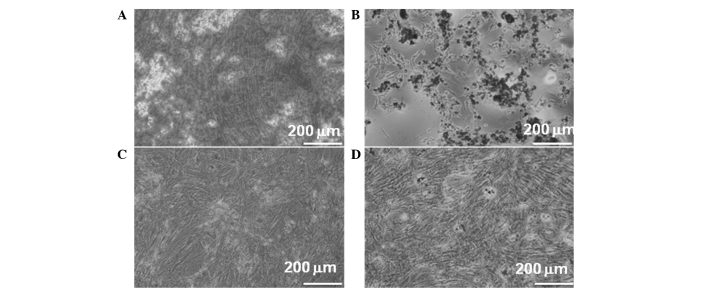
Differentiation of ASCs. ASCs were observed to differentiate toward (A) osteoblasts or (B) adipocytes in each inductive culture media following Alizarin red (A and C) and Oil Red O (B and D) staining. (C and D) No changes were observed in the cells that were not treated with inductive culture medium. ASCs, adipose tissue-derived mesenchymal stromal cells.

ASCs were tested for their capacity to differentiate toward the osteogenic and adipogenic lineages. The cells treated with osteogenic medium underwent a morphological change demonstrating calcium deposition (Fig. 1A). In the adipogenic medium, the cells may have been induced toward adipogenic differentiation as shown by the accumulation of lipid vacuoles (Fig. 1B). However, no apparent changes were observed in untreated ASCs (Fig. 1C and D).

These results demonstrated that most of the cells harbor characteristic phenotypes of ASCs to differentiate along adipogenic and osteogenic lineages.

### Effects of ASCs on alloreactively stimulated spleen cells

**Figure 2:**
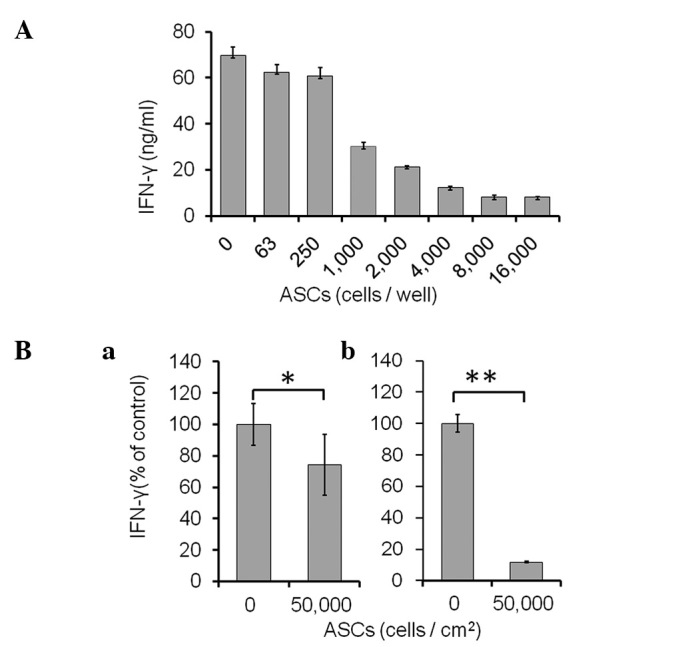
(A) Immunosuppressive effects of ASCs on alloreactively stimulated spleen cells. In each well, the indicated numbers of ASCs were mixed with 4×10^5^ spleen cells and 4×10^5^ CD3/CD28 beads. (B) Indirect (a) and direct (b) effects of ASCs on anti-CD3/CD28 bead-stimulated spleen cells were examined using Transwell chambers. (a) Spleen cells (4×10^5^) were seeded with anti-CD3/CD28 beads in the upper chamber in the presence (right column) or absence (left column) of 1.6×10^5^ ASCs seeded in the lower chamber. (b) The same number of spleen cells were seeded with anti-CD3/CD28 beads in the presence (right column) or absence (left column) of ASCs in the lower chambers. Production of IFN-γ in the supernatant (ng/ml) is indicated by the ordinate. Experiments were repeated in triplicates and results are described as the means ± standard deviation. The Student’s t-test was used to test the probability of significant differences between samples. ^*^P<0.05 and ^**^P<0.01. ASCs, adipose tissue-derived mesenchymal stromal cells; IFN-γ, interferon-γ.

It has been identified that MSCs mainly control the Th1 response (25). Among the acute inflammatory molecules, the current study focused on IFN-γ, as this cytokine is a major product of Th1 cells, which reduces the Th2 phenotype and stimulates several key functions to activate macrophages and anti-tumor reaction (26). Our preliminary experiments revealed that the production of IFN-γ by the anti-CD3/CD28 bead-stimulated spleen cells was significantly upregulated by 48 h and the elevated levels continued until 96 h (data not shown). Therefore, spleen cells (4×10^5^) and anti-CD3/CD28 beads (Invitrogen Life Technologies) were co-cultured with ASCs (0–16,000 cells/well) for 48 h (Fig. 2A). As shown in Fig. 2A, the production of IFN-γ was markedly suppressed by the ASCs in a dose-dependent manner. ASCs (n=8,000) showed maximal suppression and the suppressive level was unchanged up to 16,000 ASCs.

In addition to the direct co-culture assay, Transwell assays were performed to determine whether cell-to-cell contact was necessary for the suppression of activated spleen cells by ASCs (Fig. 2B). ASCs were plated in the lower wells; Transwell inserts containing the anti-CD3/CD28 beads and spleen cells were placed over each well. After 48 h, the volume of secreted IFN-γ was reduced to 74.4±19.4% of control (without ASCs; P<0.05) in contactless Transwell culture (Fig. 2Ba). However, the concentration of secreted IFN-γ was markedly reduced to 11.8±0.3% (P<0.01) in the direct co-culture (Fig. 2Bb).

The results revealed that ASCs require direct cell-to-cell contact to maximally suppress IFN-γ production by activated spleen cells. However, even in the absence of cell contact, ASCs partially suppressed the IFN-γ production via one or more secreted factors.

### CD69^+^ activated spleen T cells in the presence and absence of ASCs

**Figure 3:**
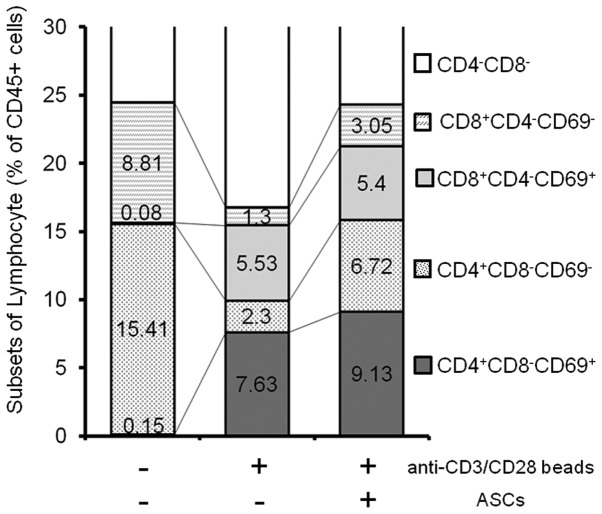
Flow cytometric analysis of T-cell subsets in CD45^+^ spleen cells. Spleen cells (4×10^5^) were cultured in the presence or absence of 4×10^5^ anti-CD3/CD28 beads and ASCs for 12 h. The cells were harvested, then FACS analyses were performed using anti-CD45 (PerCP-Cy™5.5), anti-CD4 (PE), anti-CD8 (FITC) and anti-CD69 (APC) antibodies. The percentage of each cell subpopulation (denoted on the right side) among the CD45^+^ total lymphocytes are indicated. ASCs, adipose tissue-derived mesenchymal stromal cells.

To investigate whether the activation status of T cells is changed by ASCs or not, the expression of CD69 on stimulated spleen cells was analyzed by flow cytometry (Fig. 3). CD69 is the earliest inducible cell surface glycoprotein acquired during lymphoid activation, which is involved in lymphocyte proliferation and function (27). In order to select all leukocytes, CD45^+^ cells were first gated (100%). CD69 positive/negative fractions among CD4^+^/CD8^−^ and CD4^−^/CD8^+^ T subsets were compared. The results are summarized in Fig. 3. No CD69^+^ cells were detected in CD45^+^ un-stimulated spleen cells. Following stimulation with the anti-CD3/CD28 beads, CD69^+^ populations emerged in the CD4^+^/CD8^−^ and CD4^−^/CD8^+^ T subsets, as 7.6% and 5.5%, respectively. In the presence of ASCs, the production of IFN-γ was greatly suppressed; however, certain populations of CD69^+^ continued to exist in the CD4^+^/CD8^−^ and CD4^−^/CD8^+^ subsets, as 9.1% and 5.4%, respectively.

These results suggested that the suppressive effects of ASCs on the activated spleen cells did not interfere with the signaling loop mediated by T cell receptors.

### Role of MHC class I molecules on the immunosuppressive function of ASCs

**Figure 4:**
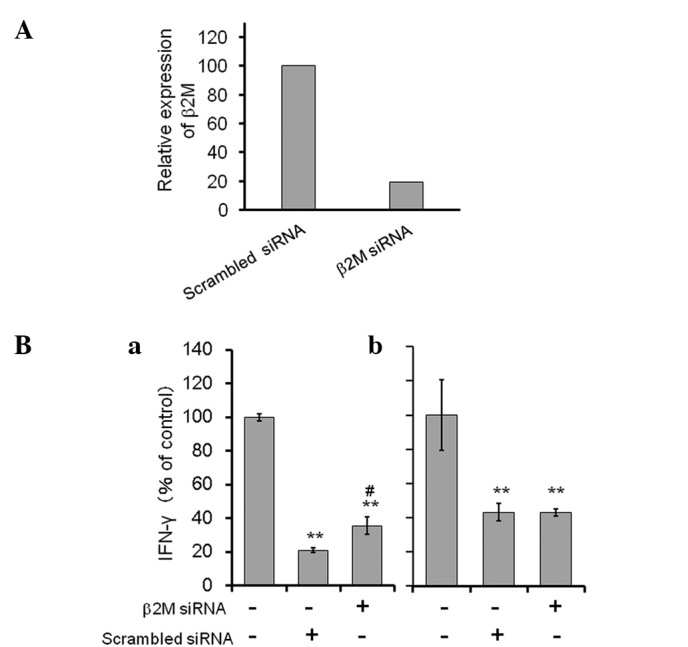
(A) Suppression of endogenous β2M transcripts in ASCs transfected with β2M-specific siRNA. The expression of endogenous β2M mRNA in siRNA-transfected ASCs was confirmed by semi-quantitative PCR. The relative expression level of β2M mRNA was normalized as a ratio to that of RPS5-mRNA. Results are described as a mean of duplicate experiments. (B) IFN-γ production by stimulated spleen cells in the presence or absence of siRNA-transfected ASCs. Anti-CD3/CD28-stimulated spleen cells were directly (a), or indirectly (b) co-cultured with β2M-specific or control (scrambled) siRNA-transfected ASCs. Relative IFN-γ levels in the supernatant are indicated by the ordinate. Experiments were repeated twice with good reproducibility. Results from a representative experiment are summarized. Values in more than triplicates were described as the mean ± standard deviation. The Student’s t-test was used to test the probability of significant differences between samples. ^#^P<0.05 β2M siRNA vs. scrambled siRNA; ^**^P<0.01 β2M siRNA or scrambled siRNA vs. control (without ASCs). β2M, β2 microglobulin; ASCs, adipose tissue-derived mesenchymal stromal cells; RPS5, ribosomal protein S5; IFN-γ, interferon-γ.

It has been identified that the immunosuppressive effects of MSCs involve a nonclassical MHC, HLA-G (28,29). HLA-G is expressed in membrane-bound and soluble isoforms (29). In order to examine the association of MHC molecules and the suppressive effects of mouse ASCs, knockdown of endogenous β2M in ASCs was performed, as the molecule is a component of MHC class I molecules, and also necessary for cell surface expression of MHC class I molecules and stability (30). As shown in Fig. 4A, β2M siRNA-transfected ASCs expressed a lower level of endogenous β2M mRNA (<20%) compared with that of the non-specific scrambled RNA-transfected cells.

In the direct co-culture of the anti-CD3/CD28-stimulated spleen cells and non-specific scrambled RNA-transfected ASCs, the level of IFN-γ production was considerably reduced (to 21% of the control). By contrast, IFN-γ production was significantly recovered (to 36% of the control) in the co-culture using β2M siRNA transfectants. Furthermore, in the contactless co-culture using a Transwell, the level of IFN-γ produced by the spleen cells was unchanged between those treated with β2M siRNA-transfected ACSs and those treated with control siRNA transfected ASCs.

The results suggest that the suppressive function of ASCs on spleen cells is directly mediated by an MHC class I complex.

## Discussion

In the present study, it was demonstrated that mouse ASCs markedly suppressed IFN-γ production by anti-CD3/CD28 bead-stimulated spleen cells; however, certain populations of CD69^+^ existed in CD4^+^/CD8^−^ and CD4^−^/CD8^+^ subsets, even in the presence of ASCs. This observation is similar to that previously reported for a co-culture with BM-derived MSCs, where proliferation was significantly reduced, while the expression of activation markers, CD25 and CD69, was unchanged in anti-CD3/CD28-stimulated T cells (31). These results suggest that the suppressive effects of ASCs on the activated spleen cells did not interfere with the signaling loop mediated by T cell receptors.

The results of the Transwell assay in the present study demonstrated that in the absence of cell contact, ASCs partially suppressed IFN-γ production, possibly via one or more secreted products. Several factors have been implicated in the immunomodulatory effects of MSCs, including PGE_2_, TGF-β1, IDO (15,16) and nitric oxide (NO) (30). However, in the present study, the results obtained with the Transwell system demonstrated that ASCs require direct cell-to-cell contact to maximally suppress the activated spleen cells.

NO is known as one of the major mediators of T-cell suppression. T-cell-MSC contact is also critical for the efficient production of NO from MSCs (31), suggesting a dynamic cross talk, including direct cell-cell contact between MSCs and lymphocytes, is required for these immunosuppressive effects. It has been reported that BM-derived MSCs express HLA-G protein, a non-classical HLA class I molecule, and the molecule has an immunosuppressive function by reducing lymphocyte proliferation (29). Stable expression of HLA-G1 has been shown to enhance the immunosuppressive effects of human ASCs (32). Furthermore, inhibitors of mevalonate synthesis have demonstrated the ability to downregulate the expression levels of adhesion molecules, including HLA class I, resulting in the prevention of immunosuppressive effects of BM-derived MSCs (33). Therefore, we have attempted to knockdown the endogenous expression of mouse MHC class I molecules, including H2-D1, H2-K1, H2-Ke and H2-Ke6 in mouse ASCs using siRNAs; however, the immunosuppressive effects of the ASCs were unaffected (data not shown). Following this, in the present study, the expression of endogenous β2M was knocked down in ASCs because this molecule is a universal component of MHC class I complexes necessary for cell surface expression and stability of MHC class I molecules (33). The results revealed that the immunosuppressive effects of ASCs on activated spleen cells were significantly, but only partially alleviated in β2M siRNA-transfected ASCs. In addition, in the contactless co-culture using a Transwell, the immunosuppressive effects of ASCs were unaffected regardless of the level of endogenous β2M, which was significantly reduced by siRNA, suggesting that the effects of β2M are not mediated merely by soluble factors. These results suggest that one or more additional MHC class I molecules may be responsible for the immunomodulatory functions of mouse ASCs, or that a combination of a number of adhesion molecules is essential to confer the function to the cells.

In conclusion, in the direct co-culture, the suppressive function of mouse ASCs on spleen cells is partially mediated by an MHC class I complex. The results of the present study may provide a novel insight for further analysis of the immunomodulatory mechanisms in MSCs.
